# Phosphorus-doped T-graphene nanocapsule toward O_3_ and SO_2_ gas sensing: a DFT and QTAIM analysis

**DOI:** 10.1038/s41598-024-54110-z

**Published:** 2024-02-12

**Authors:** Mohammad Tanvir Ahmed, Abdullah Al Roman, Debashis Roy, Shariful Islam, Farid Ahmed

**Affiliations:** 1 Department of Physics, Jashore University of Science and Technology, Jashore, 7408 Bangladesh; 2 Department of Physics, Jahangirnagar University, Dhaka, 1342 Bangladesh

**Keywords:** T-graphene, DFT, Adsorption, Toxic gas, Quantum dot, Chemical physics, Density functional theory, Carbon nanotubes and fullerenes, Quantum dots

## Abstract

Tetragonal graphene nano-capsule (TGC), a novel stable carbon allotrope of sp^2^ hybridization is designed and doped with phosphorus (P) to study the O_3_ and SO_2_ gas sensitivity via density functional theory calculation. Real frequencies verified the natural existence of both TGC and P-doped TGC (PTGC). Both TGC and PTGC suffer structural deformations due to interaction with O_3_ and SO_2_ gases. The amount of charge transfer from the adsorbent to the gas molecule is significantly greater for O_3_ adsorption than SO_2_ adsorption. The adsorption energies for TGC + O_3_ and PTGC + O_3_ complexes are − 3.46 and − 4.34 eV respectively, whereas for TGC + SO_2_ and PTGC + SO_2_ complexes the value decreased to − 0.29 and − 0.30 eV respectively. The dissociation of O_3_ is observed via interaction with PTGC. A significant variation in electronic energy gap and conductivity results from gas adsorption which can provide efficient electrical responses via gas adsorption. The blue/red shift in the optical response proved to be a way of detecting the types of adsorbed gases. The adsorption of O_3_ is exothermic and spontaneous whereas the adsorption of SO_2_ is endothermic and non-spontaneous. The negative change in entropy verifies the thermodynamic stability of all the complexes. QTAIM analysis reveals strong covalent or partial covalent interactions between absorbent and adsorbate. The significant variation in electrical and optical response with optimal adsorbent-gas interaction strength makes both TGC and PTGC promising candidates for O_3_ and SO_2_ sensing.

## Introduction

Environmental contamination caused by industrial wastes and other byproducts has increased as a result of rapid industrialization. Many dangerous gases, including those produced by motorized traffic, power plants, industry, biological waste, and other sources, are present in the environment^1^. These gases include CH_4_, CO, SO_2_, CO_2_, NO, NH_3_, CH_3_OH, H_2_S, O_3_, PH_3_, and COCl_2_^2–4^. The well-known air contaminant ozone (O_3_) has negative effects on the mucosa in the eyes and respiratory tissues. The lung is the primary target of tropospheric ozone in humans, which has detrimental effects on bodily functions^5^. Another common type of air pollution is sulfur dioxide (SO_2_), which is colorless, corrosive, and highly excitant. The interaction of SO_2_ with the surrounding air can cause several concerns to human health as well as harmful effects on the environment^4^. Monitoring these dangerous gases is crucial for creating a healthier living environment, which is what spurred the advancement of innovative techniques for sensing these gases. A sensing material demonstrates an alteration in resistivity/conductivity due to charge transfer between the adsorbent and gas molecules^6,7^.

The numerous bonding modes of carbon (C) atoms, which are widely dispersed in nature, allow them to form a broad variety of allotropes with distinct physical and chemical characteristics, including diamond carbon nanotubes, fullerene, graphene, and so on^8^. However, for toxic gas detection, pure graphene is too chemically inert and insensitive to be of much value^2^, which led the researchers to study the sensitivity of various allotropes of C in both pristine and doped states for gas sensing applications^8–15^. P-doped graphene showed strong chemisorption of SO_2_, NO_2_, O_2_, etc.^14^. According to the theoretical investigation of Yu et al., O_3_ gas decomposes to O_2_ gas via interaction with P- and N-doped graphene^10^. Pt-doped graphene showed strong adsorption of O_3_ compared to SO_2_^15^.

Fullerene showed a high sensitivity toward H_2_S, CH_4_, C_3_H_8_, etc. toxic gases in theoretical and experimental investigations^13^. The presence of several gases, e.g., NO_2_, NH_3_, and so on can alter the conductivity of carbon nanotube (CNT) significantly^16^. The sensitivity of CNT for NH_3_, NO_2_, etc. toxic gases has enhanced when decorated with metal^11,12^. Compared to all other anticipated graphene variants, including the newly produced graphene and graphdiyne, tetragonal graphene (T-graphene) is thermodynamically substantially more stable^17^. Very few studies on T-graphene's applications have been reported yet. T-graphene has shown high potential for gas sensing and ion storage applications^8,18,19^. Xie et al. reported T-graphene to be a potential candidate for NO and NO_2_ detection^8^. Li-doped T-graphene is reported to be a promising material for H_2_ adsorption and storage^19^. Liu et al. also reported that Li doping can improve the CO detection sensitivity of T-graphene^20^.

Here, we have designed and optimized a new geometries, T-graphene capsule (TGC) quantum dot (QD) and P-doped TGC (PTGC) QD using density functional theory (DFT) calculations. The adsorption of O_3_ and SO_2_ toxic gases on both TGC and PTGC has been studied for gas sensing applications. The variation in structural, electronic, optical, and thermodynamic properties due to the interaction of TGC and PTGC with selected gas molecules has been investigated. The nature of the adsorbent-adsorbate interaction has been understood via the quantum theory of atoms in molecules (QTAIM) analysis. Since TGC is a novel geometry of carbon, the gas-sensing application of TGC and PTGC has not been studied yet.

## Computational details

The nanocapsules of TGC and PTGC QDs are designed via “GaussView 06” and optimized to stable ground state energies by DFT calculation in “Gaussian 09W” software. For geometry optimization, we used the dispersion-corrected B3LYP-D3 functional with the 6-31G(d) basis set which can provide a good estimation of adsorption energies in the study^3^. The energy and frequency calculations were performed to understand the optical response and dynamic stability of the geometries. For a better estimation of the energy gap, the HSEH1PBE hybrid functional with LanL2DZ basis set was employed^21^. The stable configurations of the adsorbent + gas complex were chosen using the Adsorption Locator module^22,23^. The adsorption energy (${E}_{ads}$) of the adsorbent + gas complexes were calculated via Eq. (1),

1 $$E_{ads} = E_{complex} - E_{capsule} - E_{gas} ,$$

where, ${E}_{complex}, {E}_{capsule},$ and ${E}_{gas}$ represent the energy of the complex structures, capsules, and gas molecules respectively^24,25^. The basis set superposition error (BSSE) in the electronic structure of molecules occurs when orbitals are approximated using the expansion of basis functions, and this has to be examined. The corresponding energy of BSSE (${E}_{BSSE}$) were calculated via counterpoise method^26,27^. The adsorption energies of the complexes can be corrected using the following equation^3,28^.

2 $$E_{ads\_BSSE} = E_{ads} + E_{BSSE} ,$$

where ${E}_{ads\_BSSE}$ represents the BSSE-corrected adsorption energy.

The cohesive energy (*E*_*C*_) of the adsorbents was estimated by Eq. (3)^29^**,**

3 $$E_{C} = \frac{{E_{Adbnt} - \mathop \sum \nolimits_{x} n_{x} E_{x} ,}}{{\mathop \sum \nolimits_{x} n_{x} }},$$

where, ${{\text{E}}}_{{\text{Adbnt}}}, {E}_{x},\mathrm{ and }\,{n}_{x}$ represent the adsorbent energy, energy of individual (*x*) species, and number of *x* species. The Mulliken charges (MC), electrostatic potential map (EPM), partial density of states (PDOS), highest occupied molecular orbital (HOMO), lowest unoccupied molecular orbital (LUMO), and energy gap of the geometries were studied to understand the electronic characteristics of the structures. The vital electronic parameters and global indices are calculated by the following set of equations^30–32^.

4 $$E_{f} = \frac{1}{2}\left( {E_{H} + E_{L} } \right),$$

5 $$E_{g} = E_{L} - E_{H}$$

6 $${\Upsilon } = \frac{{E_{g} }}{2},$$

7 $${\Omega } = \frac{{E_{L} + E_{H} }}{2},$$

8 $${\uplambda } = \frac{1}{2\Theta },$$

9 $${\upxi } = \frac{{{\upnu }^{2} }}{2\Theta },$$

where *E*_*L*_*, E*_*H*_*, E*_*f*_*, E*_*g*_*, *$\Upsilon ,\Omega$*, λ,* and *ξ* represent the HOMO’s energy, LUMO’s energy, fermi energy, energy gap (HOMO–LUMO gap), global hardness, chemical potential, global softness, and electrophilicity index, respectively.

The variation in thermodynamic parameters i.e., enthalpy (H), Gibbs free energy (G), and entropy (S) can be calculated from the following relations^21^.

10 $$\Delta {\Psi } = {\Psi }_{complex} - {\Psi }_{capsule} - {\Psi }_{gas} ,$$

11 $$\Delta S = \frac{1}{T}\left( {\Delta H - \Delta G} \right),$$

where, ${\Psi }_{complex}$, ${\Psi }_{capsule}$, and ${\Psi }_{gas}$ represent H/G of the complex structures, the adsorbent, and the gas molecules. $\Delta\Psi$ denotes enthalpy variation (∆H) or free energy variation (∆G). The nature of the gas-adsorbent interactions was understood via QTAIM analysis using the AIMALL package^33^.

## Results and discussion

### Geometry analysis

**Figure 1:**
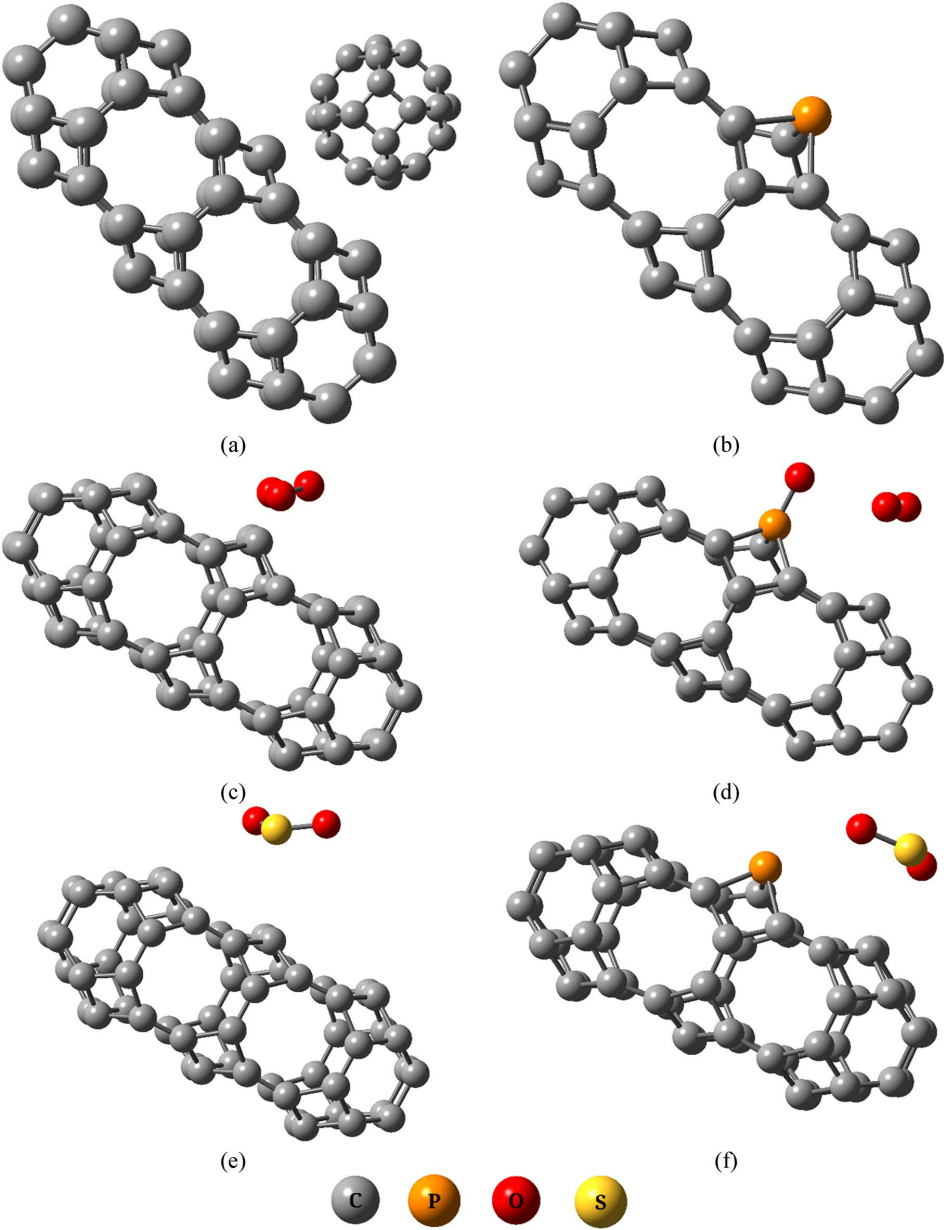
Optimized geometries of (**a**) TGC, (**b**) PTGC, (**c**) TGC + O_3_, (**d**) PTGC + O_3_, (**e**) TGC + SO_2_, and (**f**) PTGC + SO_2_.

The designed T-graphyne capsule (TGC) and P-doped T-graphyne capsule (PTGC) contain a total of 56 atoms **(**Fig. 1) possessing cohesive energies of − 8.42 and − 8.35 eV respectively suggesting a strong binding of the molecules. The average C–C bond length of the TGC QD is analogous to that of graphene, T-graphene, and CNT^34,35^. P-doping results in a significant structural deformation due to the higher atomic radius of P. Table 1 shows the variation in bond lengths between elements due to the adsorption of O_3_ gases. The values in the parenthesis represent the bond lengths before adsorption. The adsorption of O_3_ results in a significant deformation of TGC proven by the excessive changes in bond lengths. On the other hand, comparatively smaller variation in C–C bond lengths is observed due to PTGC + O_3_ interaction. However, the C–P bond lengths change to a great extent after adsorption. It is observed that the O_3_ molecule dissociates into O_2_ and oxygenates PTGC. The SO_2_ gas molecule shows lesser interaction with TGC and PTGC compared to O_3_. Very slight deformation is observed in the molecules via nominal variation in C–C, C–P, and S=O bond lengths. SO_2_ does not reveal any dissociation of molecules like O_3_. The SO_2_ gas molecule shows lesser interaction with TGC and PTGC compared to O_3_. Very slight deformation is observed in the molecules via nominal variation in C–C, C–P, and S=O bond lengths. SO_2_ does not reveal any dissociation of molecules like O_3_.

**Table 1:** Average bond lengths (Å) of the structures.

Structures	C–C	C–P	O–O	S=O
TGC	1.416	–	–	–
TGC + O_3_	1.474	–	1.448 (1.264)	–
TGC + SO_2_	1.420	–	–	1.466 (1.464)
PTGC	1.422	1.862	–	–
PTGC + O_3_	1.425	1.831	1.214	–
PTGC + SO_2_	1.421	1.864	–	1.465

### Adsorption analysis

The adsorption energy (${E}_{ads}$) represents the amount of energy required to separate the adsorbate from the adsorbent surface. Both the adsorbents show exothermic adsorption of the selected gas molecules. O_3_ shows very strong adsorption in both TGC and PTGC quantum dots. Although, SO_2_ shows weaker interaction than O_3_, the adsorption energies are still suitable for sensing application. The adsorption energies for O_3_ are significantly higher on the selected adsorbents than on B-doped graphene^36^, Pt-decorated graphene^15^, B_12_N_12_ nanocage^37^, and MoS_2_^38^. Whereas TGC and PTGC show comparatively stronger adsorption of SO_2_ than B-doped graphene^36^, terthiophene^39^, B_12_N_12_ nanocage^37^, and B_12_P_12_ nanoclusters^40^ adsorbents.

One of the vital characteristics of the adsorbent for sensing applications is the recovery time ($\tau$). The recovery time can be calculated from Eq. (12)^2,3^.

12 $$\tau = \frac{1}{{f_{r} }}e^{{ - \frac{{E_{ads} }}{{K_{b} T}}}} ,$$

where, ${f}_{r}$ is the frequency of the irradiated electromagnetic (EM) wave. In experiments, the UV radiation (${f}_{r}$ =10^12^–10^14^ Hz) is used for regenerating adsorbents. The temperature (*T*) of 298 K is used in this study.

The TGC + SO_2_ and PTGC + SO_2_ complexes show a recovery time of 2.7 ns and 3.23 ns suggesting a fast recovery via UV irradiation at room temperature makes both adsorbents suitable candidates for SO_2_ adsorption. On the other hand, due to high adsorption energies, both TGC + O_3_ and PTGC + O_3_ complexes possess a high recovery time (Table 2). The recovery time remains impractical upon X-ray irradiation at 600 K temperature suggesting that the adsorbents cannot be recoverable via the conventional irradiation technique. In this circumstance, the adsorbents may regenerate through chemical reactions. Some of the commonly used regenerating agents e.g., HCl, NaOH, HNO_3_, NaHCO_3_, etc. may show better performance in recovering both adsorbents which require further investigations^21,41^.

**Table 2:** Adsorption energy and recovery time of the studied complexes.

Complexes	E_ads_ (eV)	E_ads_BSSE_ (eV)	Recovery time (s)
TGC + O_3_	− 3.46	− 3.19	7.41 × 10^39^
TGC + SO_2_	− 0.29	− 0.20	2.70 × 10^–09^
PTGC + O_3_	− 4.34	− 4.28	1.47 × 10^58^
PTGC + SO_2_	− 0.30	− 0.21	3.23 × 10^–09^

### Vibrational properties

**Figure 2:**
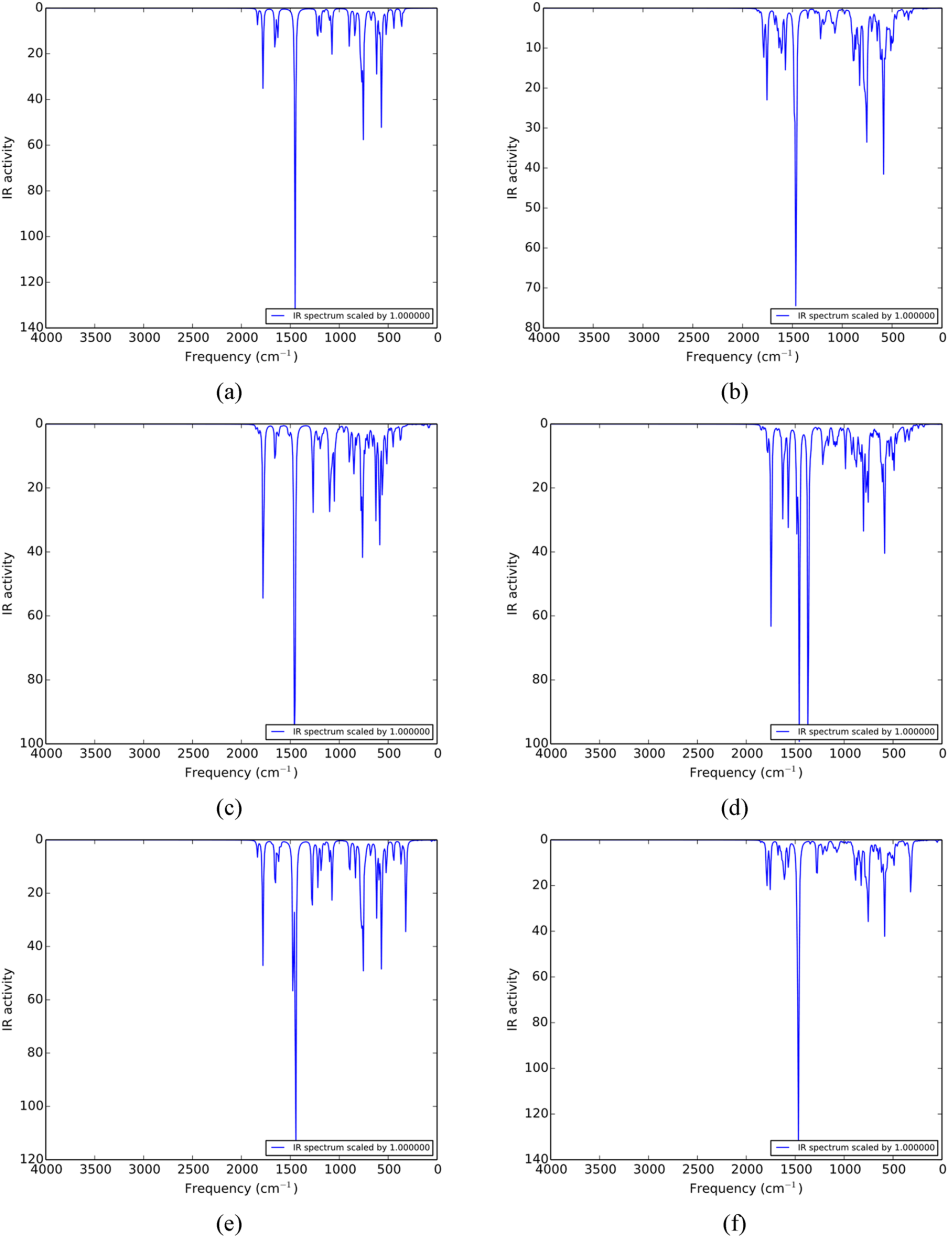
IR spectra of (**a**) TGC, (**b**) PTGC, (**c**) TGC + O_3_, (**d**) PTGC + O_3_, (**e**) TGC + SO_2_, and (**f**) PTGC + SO_2_ structures.

The frequency analysis reveals the vibrational modes in the molecular systems along with their dynamic stability. The TGC, PTGC and all their complexes possess real frequencies (Fig. 2) signifying all the geometries are stable and can exist in nature^42,43^. The IR peaks near 1528–1539 cm^−1^ indicate C–C stretching in the normal direction to the capsule length, whereas the peak near 1747 cm^−1^ represents C–C stretching along the capsule length. A P–C vibration of the PTGC molecule is observed near 1290 cm^−1^. Through the adsorption of toxic gases, a slight variation in the peak position is observed due to the structural deformation. In the TGC + O_3_ complex, O–O–O bending, O–O asymmetric stretching, O–O symmetric stretching and C–O stretching are observed at 633, 727, 916, and 1025 cm^−1^, respectively. The peaks near 502 and 1129 cm^−1^ represent O=S=O bending and S=O stretching, respectively in the TGC + SO_2_ complex. On the other hand, 499 and 1127 cm^-1^ peaks indicate the O=S=O bending and S=O stretching in the PTGC + SO_2_ complex. Due to the dissociation of O_3_ in the PTGC + O_3_ complex, O=O stretching and P=O stretching is observed near 1653 and 1249 cm^−1^, respectively.

### Electronic properties

**Figure 3:**
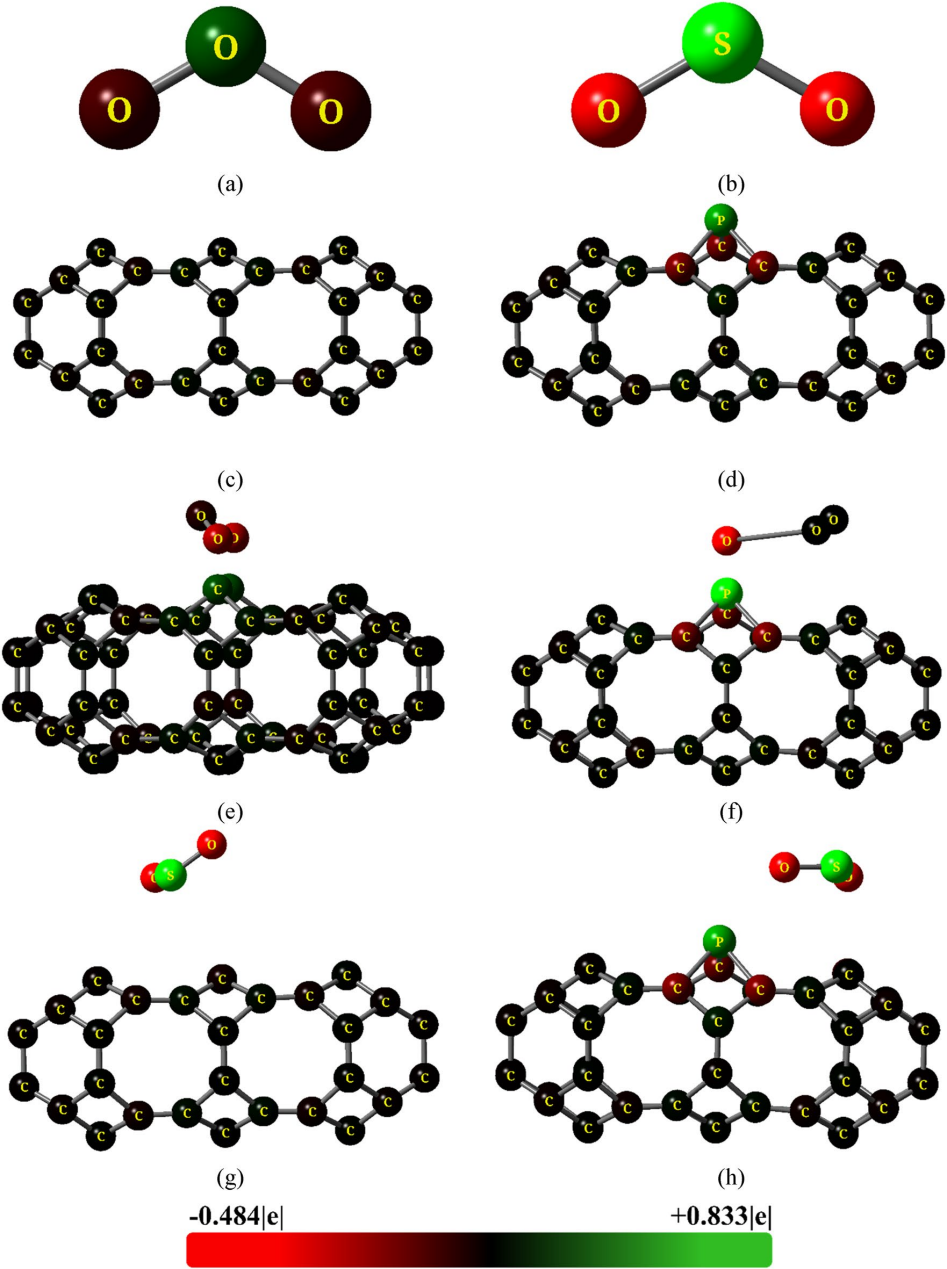
Mulliken charge distributions of (**a**) O_3_, (**b**) SO_2_, (**c**) TGC, (**d**) PTGC, (**e**) TGC + O_3_, (**f**) PTGC + O_3_, (**g**) TGC + SO_2_, and (**h**) PTGC + SO_2_.

Electronic properties can provide significant information about the sensitivity of the adsorbents toward the selected gases^44,45^. Figure 3 shows the Mulliken charge distribution of the gas molecules, adsorbents and complexes. The central Oxygen (O) atom acts as the electron donor in the O_3_ molecule, whereas in the SO_2_ molecule electrons are shifted toward O atoms due to their high electronegativity. The Mulliken charges of all carbon (C) atoms are almost neutral in the TGC structure resulting in no net charge transfer between atoms. A significant charge transfer is observed in the TGC + O_3_ complex with C atoms acting as electron donors. However, no notable change in color distribution in observed due to SO_2_ adsorption on TGC, suggesting a nominal charge displacement.

The PTGC QD showed charge asymmetry since the electronegativity of C is greater than that of P. Hence, P–C bonding electrons are displaced toward C resulting in partially negative C atoms and partially positive P atom. A strong negative charge transfer from adsorbent to O_3_ molecule is demonstrated by a significant variation in the color of P and O atoms. Two oxygen atoms almost become charge neutral whereas electrons are shifted toward the 3rd O atom from P. This significant change displacement results in a strong adsorbent-adsorbate interaction. A very slight change in charge of the P atom is observed due to the interaction with SO_2_ gas, suggesting a nominal charge transfer between absorbent and absorbate as observed in on TGC adsorbent, resulting in a weak attraction of SO_2_.

**Figure 4:**
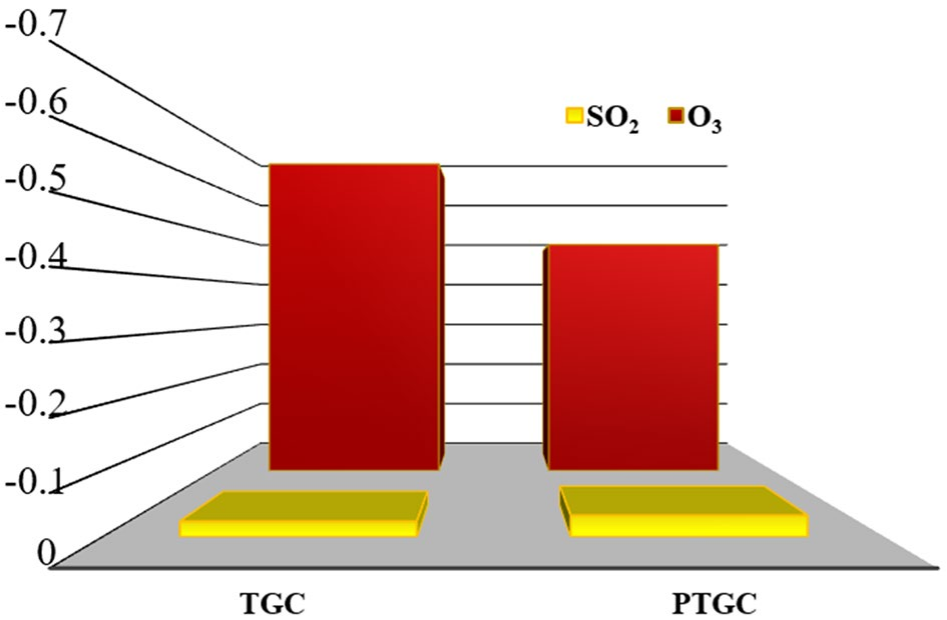
Mulliken charge transfer between adsorbent and gas molecules.

Figure 4 shows the overall charge transfer toward gas molecules via adsorbate-adsorbent interaction. The maximum charge transfer on O_3_ molecule occurs on TGC adsorbent whereas PTGC shows more charge transfer compared to TGC toward SO_2_.

**Figure 5:**
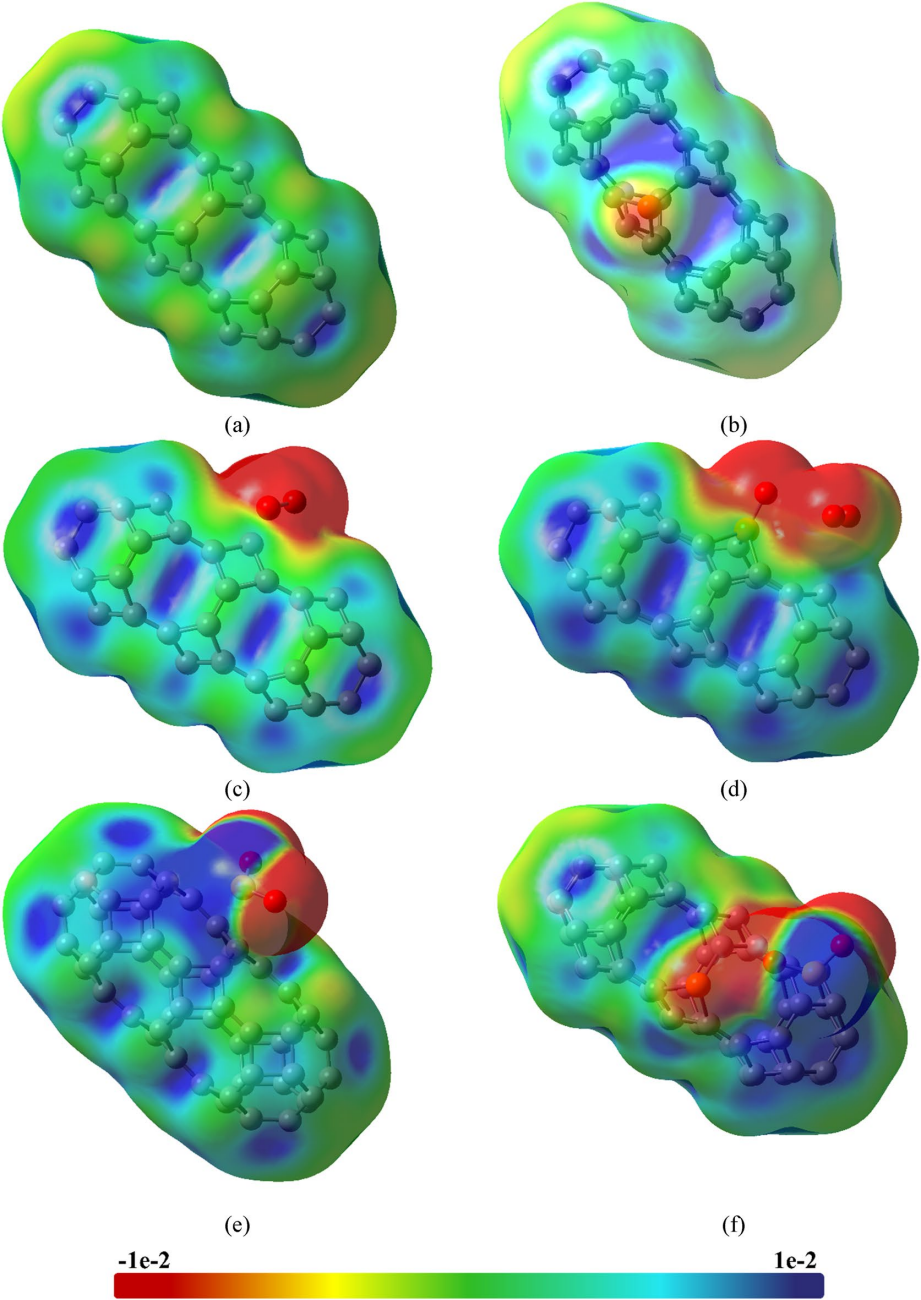
Electrostatic potential map of (**a**) TGC, (**b**) PTGC, (**c**) TGC + O_3_, (**d**) PTGC + O_3_, (**e**) TGC + SO_2_, and (**f**) PTGC + SO_2_.

Figure 5 demonstrates the electrostatic potential map (EPM) of the absorbents and complexes. is an indispensable tool for deciphering the electrostatic potential arising from the arrangement of electrons and nuclei within a molecule, thereby allowing for the identification of potential reaction sites for both electrophilic and nucleophilic attacks. The red region indicates the negative potential i.e., the electron-rich region whereas the blue region represents the electron deficit region or positive potential region. The TGC adsorbent is almost neutral resulting in a uniform distribution of negative and positive charges. On the other hand, due to the asymmetry of charge distribution in the PTGC, the P-site region becomes a comparatively stronger electrophilic attack zone. The interaction with O_3_ and SO_2_, the O-site region demonstrates to be a more electron-rich region due to the high electronegativity of the O atom. The S-site region shows comparatively positive potential due to low electron density. The noticeable color change in both TGC and PTGC upon adsorption indicates a substantial charge transfer, implying a robust adsorbent-adsorbate interaction.

**Figure 6:**
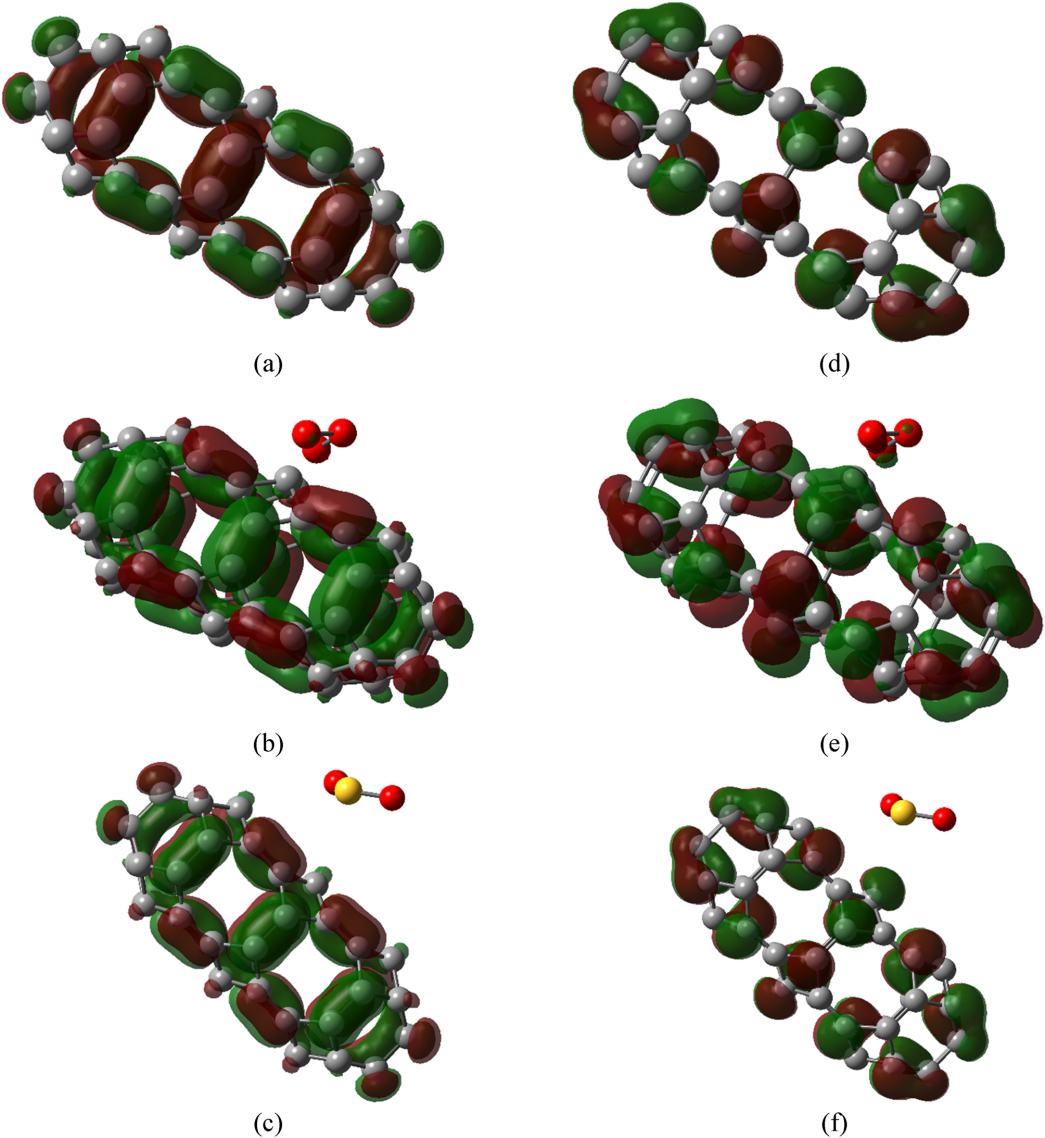
HOMOs of (**a**) TGC, (**b**) TGC + O_3_, (**c**) TGC + SO_2_, and LUMOs of (**d**) TGC, (**e**) TGC + O_3_, (**f**) TGC + SO_2_.

**Figure 7:**
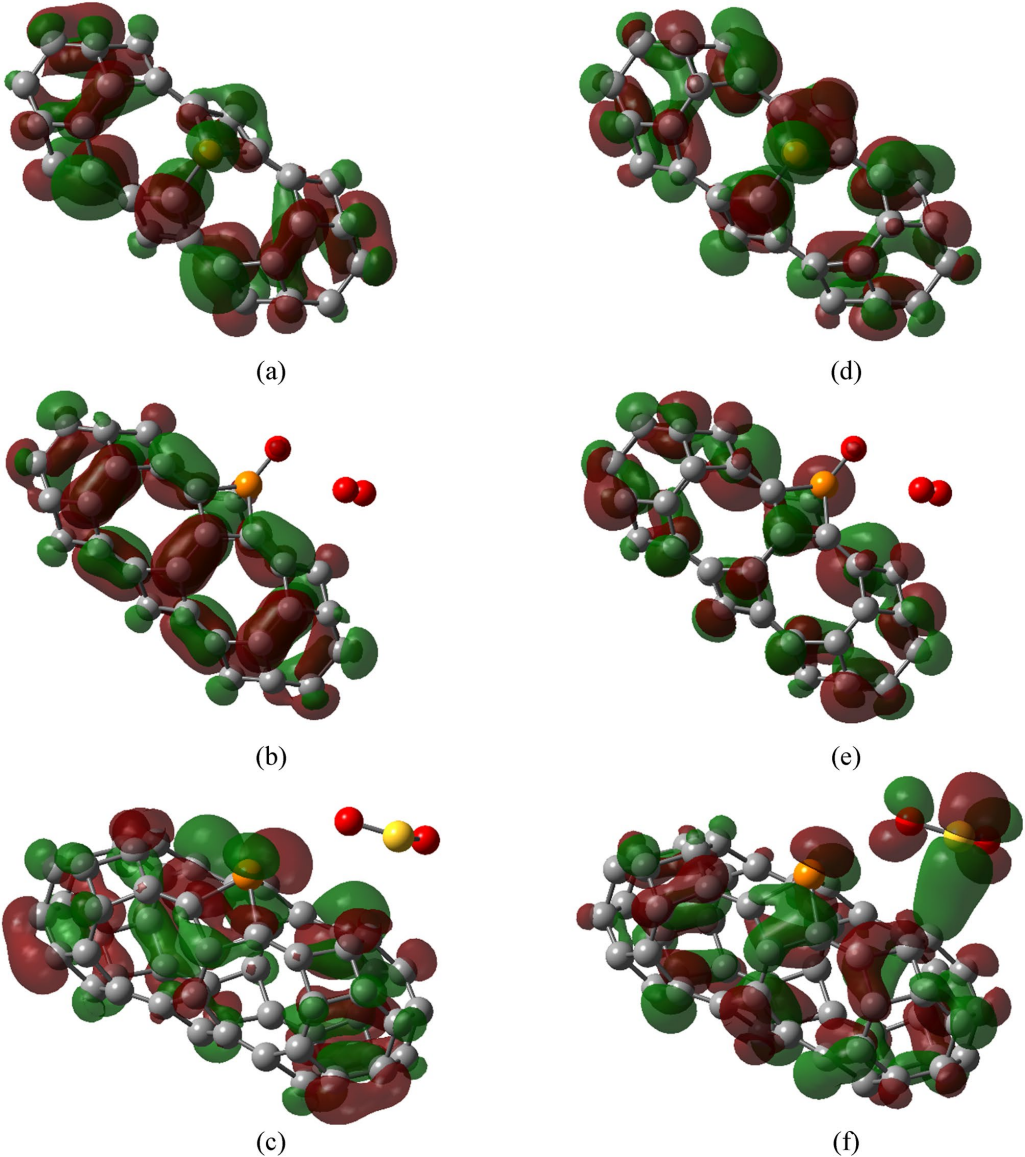
HOMOs of (**a**) PTGC, (**b**) PTGC + O_3_, (**c**) PTGC + SO_2_, and LUMOs of (**d**) PTGC, (**e**) PTGC + O_3_, (**f**) PTGC + SO_2_.

Figures 6 and 7 show the HOMOs and LUMOs TGC and PTGC along with their complexes respectively. Both HOMO and LUMO are localized in the TGC after gas adsorption. A nominal contribution to HOMO and LUMO is localized on the O_3_ molecule adsorbed on TGC. On the other hand, a significant localization of LUMO was observed on the SO_2_ molecule of the PTGC + SO_2_ complex. Both adsorbents demonstrate a significant change in HOMOs and LUMOs due to toxic gas adsorption signifying strong interaction with notable charge transfer. The energies of HOMOs and LUMOs in the geometries are presented in Table 3.

**Table 3:** E_H_, E_L_, E_g_, and φ of the structures in the unit of eV.

Structures	E_H_ (eV)	E_L_ (eV)	E_g_ (eV)	*φ* (eV)
TGC	− 6.16	− 4.98	1.18	5.57
TGC + O_3_	− 6.24	− 4.96	1.28	5.6
TGC + SO_2_	− 6.3	− 5.11	1.19	5.705
PTGC	− 4.8	− 4.18	0.62	4.49
PTGC + O_3_	− 4.87	− 3.88	0.99	4.375
PTGC + SO_2_	− 4.85	− 4.69	0.16	4.77

**Figure 8:**
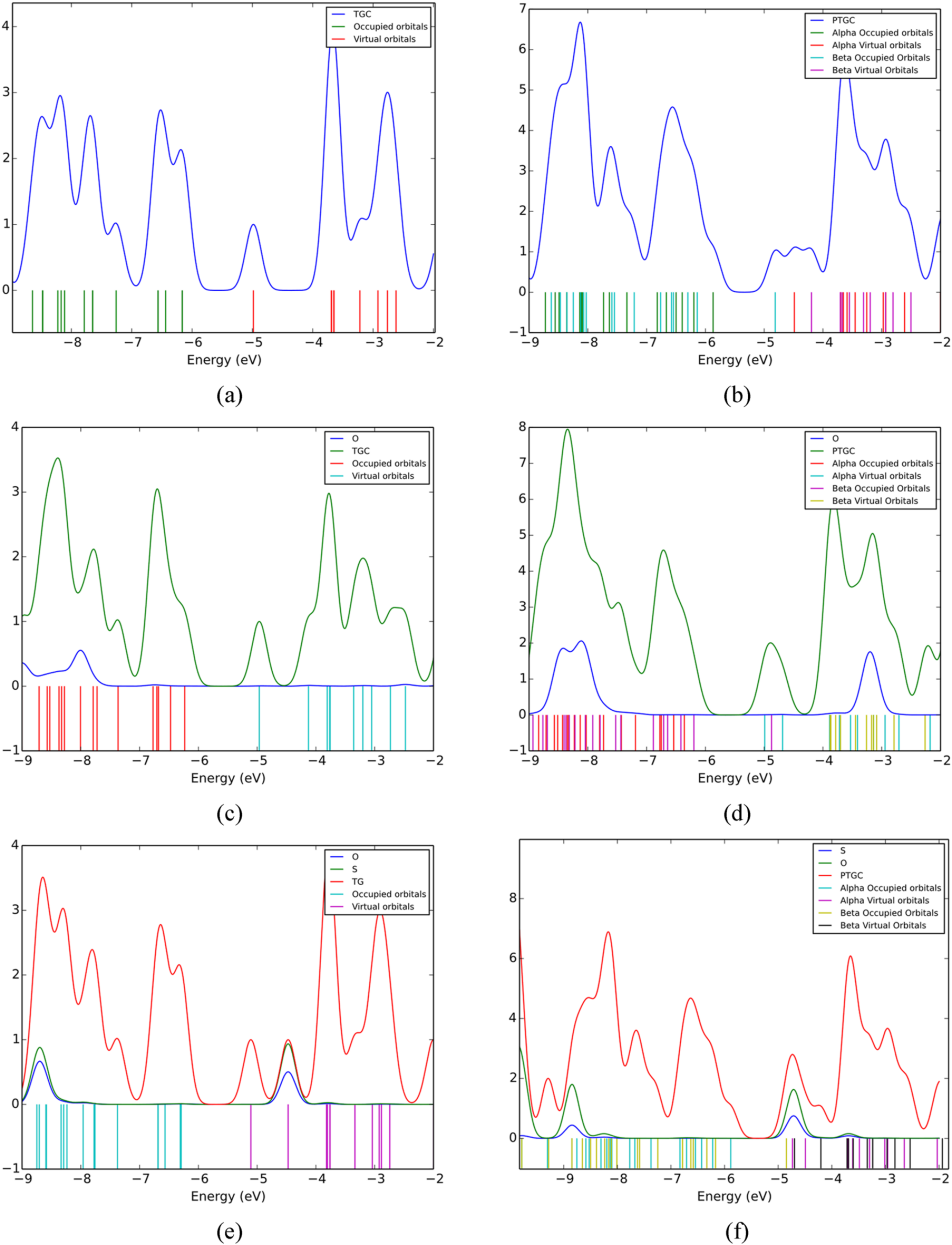
PDOS spectra of (**a**) TGC, (**b**) PTGC, (**c**) TGC + O_3_, (**d**) PTGC + O_3_, (**e**) TGC + SO_2_, and (**f**) PTGC + SO_2_ structures.

**Figure 9:**
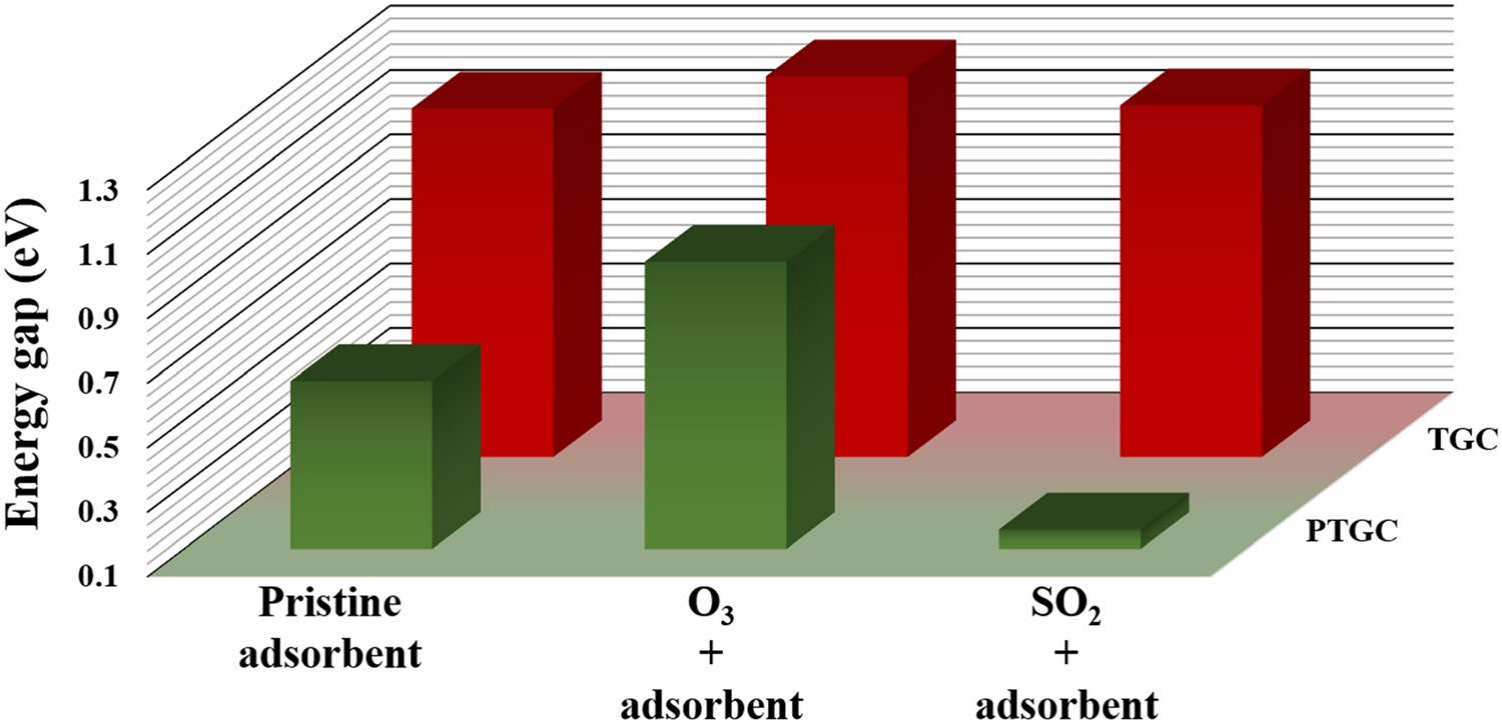
Variation of energy gap due to gas adsorption.

The PDOS spectra are analyzed for a thorough understanding of electronic properties’ variation (Fig. 8). The TGC showed an energy gap of 1.18 eV making it suitable for numerous semiconducting applications (Table 3). P-doping results in a low energy gap semiconductor through energy gap tuning. The energies of HOMOs and LUMOs of both adsorbents suffer a significant variation due to gas adsorption, resulting in an alteration of the energy gap. It is observed that the energies HOMOs of both TGC and PTGC significantly decrease after gas adsorption (Table 3), whereas the LUMO energies increase due to O_3_ adsorption but decrease through SO_2_ adsorption. The O and S atoms do not demonstrate any contribution to HOMOs and LUMOs of TGC and PTGC (except PTGC + SO_2_, where a significant orbital contribution of both O and S are observed at LUMO). The observed PDOS spectra justify the HOMO and LUMO representation in Figs. 6 and 7. Figure 9 shows the variation in the energy gap of TGC and PTGC due to the interaction with gas molecules.

The variation in the energy gap can be a result of the variation in charge distribution and structural deformation in the adsorption process^21,46^. The change in energy gap results in a variation of electronic conductivity (EC) which follows the Eq. (13).

13 $$\sigma \propto e^{{ - \frac{{E_{g} }}{{2K_{b} T}}}}$$

where, ${K}_{b}$ and $T$ represent the Boltzmann constant and absolute temperature^3^. The change in conductivity can provide a significant electrical response due to gas adsorption which makes TGC and PTGC potential materials for O_3_ and SO_2_ sensing. Through proper calibration of EC, the adsorbent can be of use in the detection of the type of adsorbed gas.

The work function (*φ*), which is the energy needed to remove an electron from the fermi level and bring it to the vacuum, is a surface-dependent characteristic of the adsorbents. The materials' conductivity is impacted by changes in work function^47^. The work function is related to the fermi energy by the following equation,

14 $$\varphi = \left| {V_{\infty + } - E_{f} } \right|$$

where, ${{V}_{\infty }}_{+}$ is the electrostatic potential of an electron far away from the adsorbent’s surface. Table 3 shows that *φ* varies significantly via gas interaction suggesting a significant change in conductivity of the adsorbents. The change in current conduction via *φ* variation can be obtained via the Richardson–Dushman relation,

15 $$J = A_{R} T^{2} e^{{ - \frac{\varphi }{{K_{b} T}}}} ,$$

where, J and A_R_ are the emitted electron density and the Richardson constant, respectively^48^.

### Global indices

Several reactivity parameters such as hardness, softness, chemical potential, and electrophilicity are calculated and their numerical values are given in Table 4. The corresponding values of Chemical hardness and electrophilicity, respectively, are small and large as compared to various carbon and boron nitride nanostructures while the chemical potential values are quite similar^49–51^. All the complexes of TGC and PTGC offer negative chemical potential value indicating their structural stability. The chemical hardness is a measure of opposition to charge transfer from a material whereas the softness signifies its opposite characteristic^52^. The hardness of TGC is increased slightly after absorption of O_3_ and SO_2_ indicating chemical stability upon gas adsorption. P doping reduces the value of hardness to about half that of its pristine structure. Although O_3_ adsorption causes PTGC to almost regain the hardness of its pristine form, SO_2_ adsorption dramatically declines the hardness and hence increases the chemical reactivity of PTGC which is also ensured by the very large value of electrophilicity.

**Table 4:** Global hardness, softness, chemical potential, and electrophilicity index of the structures.

Structures	Hardness (eV)	Softness (1/eV)	Chemical potential (eV)	Electrophilicity (eV)
TGC	0.59	0.85	− 5.57	26.29
TGC + O_3_	0.64	0.78	− 5.6	24.5
TGC + SO_2_	0.6	0.83	− 5.71	27.17
PTGC	0.31	1.61	− 4.49	32.52
PTGC + O_3_	0.5	1	− 4.38	19.18
PTGC + SO_2_	0.08	6.25	− 4.77	142.21

### Thermal properties

The thermodynamic stability of the complex structures can be understood from the change in enthalpy, Gibbs free energy, and entropy^26^. Their corresponding values after the adsorption of O_3_ and SO_2_ are tabulated in Table 5. The change in enthalpy (ΔH) upon O_3_ adsorption for both TGC and PTGC are found to be − 3.15 and − 4.07 eV/atom respectively which signify that their interactions are exothermic and the bonding between the atoms of TGC + O_3_ and PTGC + O_3_ are more stable. Similarly, the change in Gibbs free energies (ΔG) is also negative for O_3_ adsorption and positive for SO_2_ adsorption which implies that O_3_ adsorption is a spontaneous process whereas SO_2_ adsorption is a non-spontaneous process^26,53^. The interaction of SO_2_ with TGC and PTGC is endothermic with a ΔH value of 0.005 and 0.01 eV/atom respectively.

**Table 5:** Change is enthalpy, Gibbs free energy, and entropy due to adsorption.

Complexes	ΔH (eV/atom)	ΔG (eV/atom)	ΔS (eV/K)
TGC + O_3_	− 3.15	− 2.54	− 0.0020
TGC + SO_2_	0.005	0.37	− 0.0012
PTGC + O_3_	− 4.07	− 3.73	− 0.0011
PTGC + SO_2_	0.01	0.38	− 0.0012

Entropy is a measure of a molecule's freedom of motion. It is higher when the product molecules have greater freedom of motion and is less positive if the product molecules are more ordered. The change in entropy due to the adsorption of O_3_ and SO_2_ on both TGC and PTGC is negative indicating that the complexes are thermodynamically ordered and stable^21,54^.

### Optical properties

**Figure 10:**
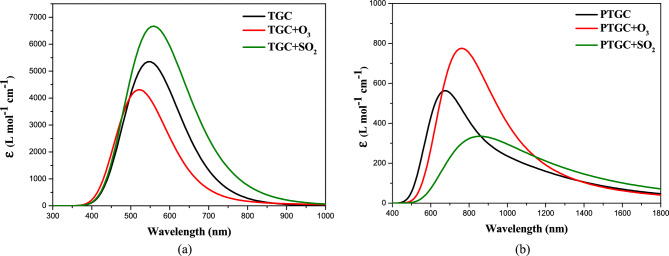
UV visible spectra of the adsorbents and complexes.

The molar absorption coefficient (*ε*), as determined through UV–visible response analysis for all structures, is presented in Fig. 10. Both pristine configurations displayed a significant absorption coefficient (AC) with a strong absorption peak in the visible wavelength region. PTGC shows strong absorption in higher wavelength regions compared to TGC, verifying the smaller energy gap of PTGC than TGC. The strong absorption in the visible wavelength region makes both TGC and PTGC potential candidates for optoelectronic research. An increased optical responsiveness at a particular wavelength is correlated with a greater AC. In both adsorbents, gas adsorption is shown to cause a significant change in the AC spectra. Along with the variation in the intensity of AC, a significant red/blue shifting of the absorption peak is observed due to the interaction with gas molecules. The reflectivity of the is related to the AC by the following equations,

16 $$Abs = C\varepsilon l,$$

17 $$Abs = - \log \left( {Tra} \right),$$

18 $$Ref = 1 - \sqrt {\left( {Tra} \right)e^{Abs} } ,$$

where *Abs*, *C*, *l*, *Tra*, and *Ref* indicate the absorbance, concentration, material thickness, transmittance, and reflectance, respectively^21^.

As a result, a red/blue shift in AC likewise produces a red/blue shift in the structure's reflectivity, changing its color; in other words, adsorbents may change color by gas adsorption. Consequently, color fluctuation may be used to identify and detect the type of toxic gas adsorbed on TGC or PTGC.

### QTAIM analysis

**Figure 11:**
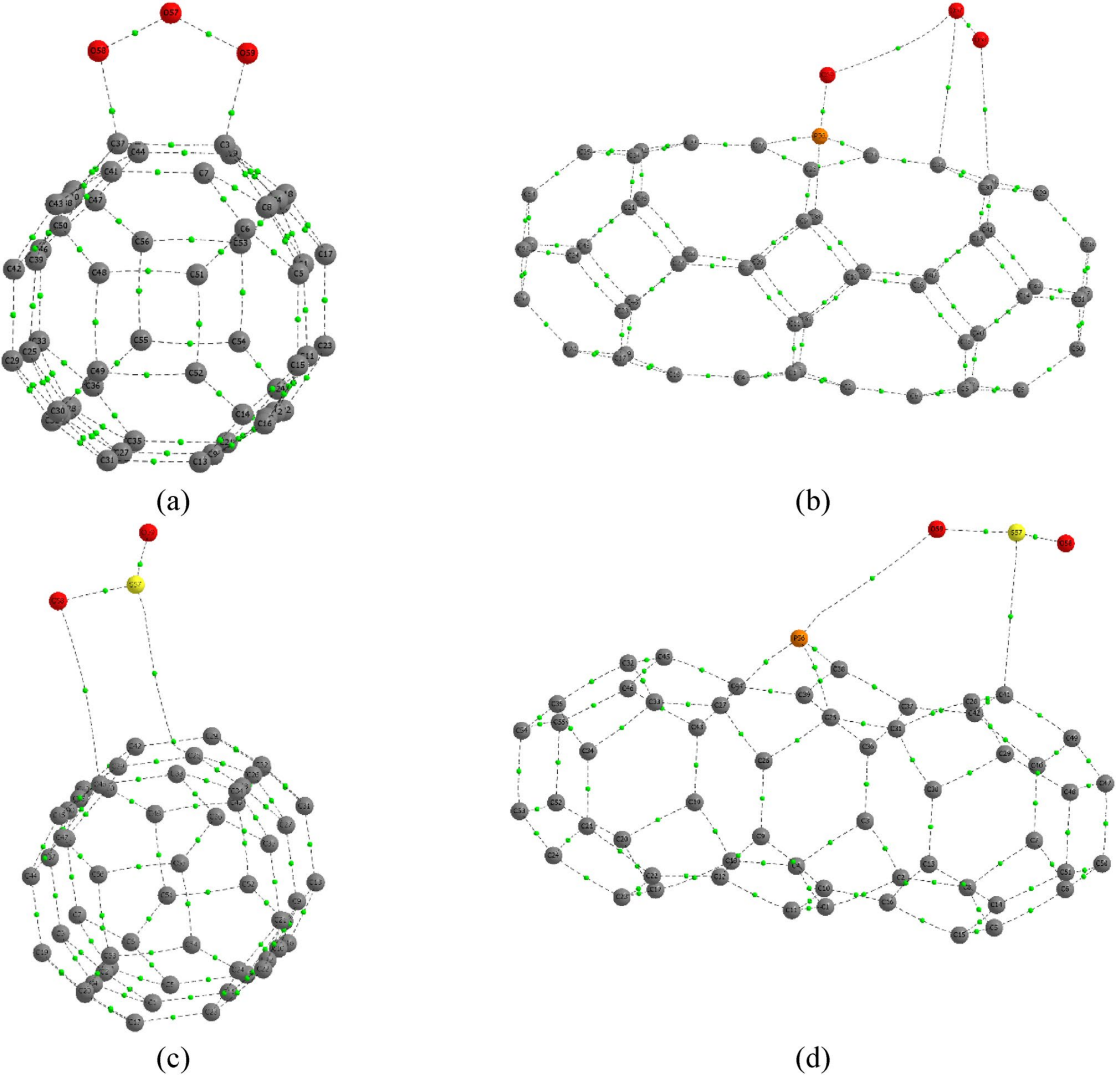
Molecular graph of the (**a**) TGC + O_3_, (**b**) PTGC + O_3_, (**c**) TGC + SO_2_ and (**d**) PTGC + SO_2_ complexes.

To gain a profound understanding of the nature and strength of molecular interactions, an in-depth Quantum Theory of Atoms in Molecules (QTAIM) analysis was performed on all complex structures. Figure 11 illustrates the molecular graph of these structures, with the green spots indicating the bond critical (BC) point. This BC point is crucial for assessing and ensuring the strength of intermolecular bonds. Hence, to characterize different types of interactions, descriptors including electron density $({\rho }_{e})$,

${\nabla }^{2}({\rho }_{e})$, electron’s kinetic energy density (ED) (${G}_{e}$), potential ED (${V}_{e}$), and total ED (${H}_{e}$) have been acquired at bond critical (BC) points (Table 6).

**Table 6:** Electron density (${\rho }_{e}$), ${\nabla }^{2}({\rho }_{e})$, electron’s kinetic ED (${G}_{e}$), potential ED (${V}_{e}$), total ED (${H}_{e}$), and $-\frac{{G}_{e}}{{V}_{e}}$ ratios in a.u. unit at BC point of intermolecular bonds.

Complexes	Bonds	${\rho }_{e}$	${\nabla }^{2}({\rho }_{e})$	${G}_{e}$	${V}_{e}$	${H}_{e}$	$-\frac{{G}_{e}}{{V}_{e}}$
TGC + O_3_	C3–O59	0.2656	− 0.5571	0.2378	− 0.615	− 0.3771	0.3867
	C37–O58	0.2658	− 0.557	0.2384	− 0.6161	− 0.3777	0.387
	O57–O58	0.2851	− 0.0306	0.2225	− 0.4527	− 0.2302	0.4915
	O57–O59	0.2865	− 0.0345	0.2236	− 0.4559	− 0.2323	0.4905
TGC + SO_2_	C46–O58	0.0023	0.0087	0.0016	− 0.0011	0.0005	1.4936
	C25–S57	0.007	0.0181	0.0035	− 0.0026	0.001	1.3861
	S57–O58	0.2738	1.2363	0.6049	− 0.9007	− 0.2958	0.6716
	S57–O59	0.2739	1.2389	0.6056	− 0.9014	− 0.2959	0.6718
PTGC + O_3_	P56–O59	0.2195	1.5584	0.5306	− 0.6716	− 0.141	0.79
	O57–O58	0.5185	− 0.6462	0.4689	− 1.0993	− 0.6304	0.4265
	O59–O57	0.0059	0.0247	0.0055	− 0.0047	0.0007	1.1536
	C31–O57	0.0047	0.0179	0.0036	− 0.0028	0.0008	1.2903
	C28–O58	0.0072	0.0235	0.0051	− 0.0042	0.0008	1.192
PTGC + SO_2_	P56–O59	0.0073	0.024	0.0053	− 0.0047	0.0007	1.1427
	C41–S57	0.0136	0.0386	0.0081	− 0.0065	0.0016	1.2469
	S57–O58	0.2741	1.2384	0.6059	− 0.9021	− 0.2963	0.6716
	S57–O59	0.2725	1.1996	0.5953	− 0.8907	− 0.2954	0.6683

The presence of BC points in a molecule validates the creation of chemical bonds between the adsorbent and gas molecules and verifies that electron density is being transferred. The atoms in a molecule bind strongly and covalently if the values both ${\nabla }^{2}({\rho }_{e})$ and ${{\text{H}}}_{{\text{e}}}$ found are negative, whereas for ${\nabla }^{2}\left({\rho }_{e}\right)>0 \,\mathrm{and }\,{{\text{H}}}_{{\text{e}}}>0,$ the interaction is weak and electrostatic^31,32^. On the other hand, the bonding nature is partially covalent for ${\nabla }^{2}\left({\rho }_{e}\right)>0 \,\mathrm{but }\,{{\text{H}}}_{{\text{e}}}<0$. The bonding type is also determined by the.

$-\frac{{G}_{e}}{{{\text{V}}}_{{\text{e}}}}$ ratio. The bond is regarded as covalent if the ratio is less than 1 (i.e.,

$-\frac{{G}_{e}}{{{\text{V}}}_{{\text{e}}}}<0.5$), while the interaction is thought to be totally non-covalent (weak electrostatic) for $-\frac{{G}_{e}}{{{\text{V}}}_{{\text{e}}}}>1$^26,47^. If $0.5< -\frac{{G}_{e}}{{{\text{V}}}_{{\text{e}}}}<1$, the bond can be identified as partially covalent.

According to the study, the interaction of the O_3_ molecule with TGC is strongly covalent resulting in a high adsorption energy. However, the dissociation of O_3_ by PTGC is proved via QTAIM analysis. The O=P bond is shown to be partially covalent, whereas the O=O of O_2_ gas is strongly covalent. The O_2_ gas interacts with the 3^rd^ O atom bonded with P via weak electrostatic force. The S=O bond is shown to be partially covalent, whereas the interaction of SO_2_ with both TGC and PTGC is non-covalent which results in comparatively low adsorption energies.

## Conclusion

Quantum dots of TGC and PTGC have been designed and optimized successfully via DFT calculations. Both structures showed real frequencies revealing the possibility of their natural existence. The sensitivity of the capsules towards O_3_ and SO_2_ toxic gases has been studied. O_3_ reveals a comparatively stronger attraction toward both TGC and PTGC than SO_2_ gas. O_3_ gas dissociates to O_2_ via interacting with PTGC. A significant structural deformation and charge transfer suggest a preferable adsorbate-adsorbent interaction for sensing applications. The high adsorption energies of O_3_ on the adsorbents result in a huge recovery time, which makes the regenerating of TGC and PTGC via the conventional irradiation process impractical. The TGC and PTGC may recover from O_3_ gas, via interacting with various regenerating agents. The charge transfer and structural deformation result in a variation of HOMO and LUMO revealing a change in the energy gap of the absorbent, which can offer an alteration of conductivity allowing the absorbents to sense the presence of toxic gases. The optical property reveals a significant shift in UV–visible spectra in the visible region due to gas adsorption, which can allow determining the nature of adsorbed gas via color change. The adsorption of O_3_ and SO_2_ are exothermic and endothermic process respectively where all the resulting complexes are thermodynamically stable. The QTAIM analysis reveals the nature of adsorbent-adsorbate interaction to be strong or partially covalent. The electronic and optical properties suggest both TGC and PTGC to be potential candidates for numerous optoelectronic applications. The strong adsorption energies with variation in conductivity and optical response make both TGC and PTGC potential adsorbent materials for O_3_ and SO_2_ gas sensing.

### Supplementary Information

Supplementary Information.

## Data Availability

Research data is shared in the “Supplementary file”.
